# Sepsis toxicity network reconstruction—Dynamic signaling and multi-organ injury: A review

**DOI:** 10.17305/bb.2025.12931

**Published:** 2025-09-02

**Authors:** Shuai Liu, Qun Liang

**Affiliations:** 1 Graduate School, Heilongjiang University of Chinese Medicine, Harbin, China; 2 Department of Critical Care Medicine, First Affiliated Hospital of Heilongjiang University of Chinese Medicine, Harbin, China

**Keywords:** Sepsis, systemic toxicity, network biology, multiple organ dysfunction, dynamic evolution of signaling pathways

## Abstract

Sepsis is a complex systemic disease in which systemic toxicity—arising from inflammation–immune dysregulation, oxidative stress, programmed cell death (apoptosis, pyroptosis, ferroptosis), and metabolic reprogramming—drives multi-organ injury. The aim of this review was to synthesize how signaling pathways evolve within and between key organs (lungs, liver, kidneys, heart) and to evaluate whether multi-omics integration and network modeling can identify critical toxic nodes and predict disease progression. We conducted a narrative review of English-language mechanistic studies published between 2015 and 2025 in PubMed, Web of Science, and Scopus, supplemented by bibliography screening, while excluding case reports, conference abstracts, and non-mechanistic work. The evidence depicts a high-dimensional systemic network that remodels over time, with early pro-inflammatory modules transitioning toward immunosuppression and organ-specific injury patterns, while inter-organ propagation is mediated by damage-associated molecular patterns (DAMPs), exosomes, and metabolites. Oxidative stress and mitochondrial dysfunction, via reactive oxygen species (ROS), couple to pyroptosis and ferroptosis to reinforce toxicity loops, and computational approaches such as dynamic Bayesian networks (DBNs) and graph neural networks (GNNs) delineate regulatory hubs and support forecasting. Therapeutic progress has concentrated on nuclear factor kappa-light-chain-enhancer of activated B cells (NF-κB), the NOD-, leucine-rich repeat and pyrin domain-containing protein 3 (NLRP3) inflammasome, and glutathione peroxidase 4 (GPX4), alongside artificial intelligence (AI)-assisted personalized toxicity maps and dynamic early-warning systems, though challenges remain in specificity, safety, and resistance. In conclusion, sepsis can be conceived as a temporally staged systemic toxicity network, and when combined with multi-omics, DBN/GNN modeling, and AI-enabled decision support, this framework offers a path toward individualized, mechanism-based care, while requiring rigorous validation to ensure clinical durability.

## Introduction

Sepsis is a systemic condition triggered by infection, characterized by a dysregulated host response that leads to progressive organ dysfunction and, in many cases, death [1, 2]. It is recognized as one of the most significant challenges in critical care medicine globally. According to a Global Burden of Disease study, approximately 49 million cases of sepsis occur annually worldwide, resulting in around 11 million deaths—accounting for nearly 20% of all global mortality [3]. Despite advancements in early recognition, prompt antimicrobial therapy, fluid resuscitation, and organ support in recent years, clinical outcomes for sepsis remain poor, particularly among patients who develop multiple organ dysfunction syndrome (MODS) in the intermediate or late stages, with mortality rates reaching 40%–60% [4]. Traditionally, sepsis has been viewed as an immune hyperactivation syndrome driven by a cytokine storm [5, 6]. However, emerging evidence from both clinical and basic research suggests that immune activation and suppression do not operate in isolation. Rather, they coexist and dynamically interact, influencing disease progression. For example, some patients exhibit a pronounced pro-inflammatory response in the early stages, indicated by elevated levels of IL-6 and tumor necrosis factor alpha (TNF-α) [7, 8], while others rapidly transition into an immunosuppressive state characterized by T cell exhaustion, impaired antigen presentation, and persistent infection [9, 10]. This clinical heterogeneity highlights that sepsis is not driven by a single pathological pathway, but rather by a complex systemic network involving immune dysregulation, metabolic reprogramming, cell death, oxidative stress, and microcirculatory disturbances [11, 12]. This evolving understanding has prompted researchers to move away from the traditional linear inflammation model, adopting perspectives focused on systemic toxicity and network regulation to redefine the pathophysiology of sepsis [13]. In this context, “dynamic evaluation” denotes the continuous, time-resolved assessment of the evolution of signaling pathways, transitions in immune status, and organ-specific responses throughout the course of sepsis. This concept underscores that pathological changes are not static but occur in a temporally staged manner, providing opportunities to identify critical intervention points.

Within this framework, sepsis is conceptualized as a high-dimensional biological network comprised of multiple signaling pathways that become destabilized and undergo reconstruction due to infection, metabolic dysregulation, and stress, ultimately resulting in organ dysfunction and structural damage. In this review, “network reconstruction” is defined in two senses: (i) the biological remodeling of signaling and metabolic circuits during disease progression and (ii) the computational and systems biology strategies (e.g., dynamic Bayesian networks [DBN] and graph neural networks [GNNs]) that model and interpret these alterations. This expanded definition emphasizes that the concept encompasses both the biological rewiring processes in sepsis and the analytical methods employed to study them. For example, classical pro-inflammatory pathways such as nuclear factor kappa-light-chain-enhancer of activated B cells (NF-κB), MAPK, and JAK-STAT are rapidly activated during the early stages of sepsis to initiate host defense responses [12, 14]. However, inadequate negative feedback regulation can lead to sustained inflammatory injury. As the disease progresses, immunosuppressive pathways—including PD-1/PD-L1, IL-10, and Suppressor of Cytokine Signaling (SOCS)—are activated, inhibiting immune cell function and resulting in a state of immune paralysis [15, 16]. Additionally, programmed cell death processes—including pyroptosis, ferroptosis, and necroptosis—along with mitochondrial dysfunction and disturbances in energy metabolism, occur concurrently across multiple organs, collectively accelerating the systemic spread of toxicity [17–19]. The complex interplay of multiple signaling pathways and biological processes complicates traditional single-factor approaches in elucidating the underlying mechanisms of sepsis. Network biology and systems toxicology offer new frameworks for investigating this condition by constructing interaction maps that illuminate key pathways, central hubs, and coordinated changes during disease progression [20, 21]. In sepsis research, particular emphasis is placed on the dynamic remodeling of signaling pathways across various time points, organs, and immune states [12, 22]. This includes the migration and distribution of distinct immune cell types within the lungs, kidneys, and liver, which contribute to both local and systemic inflammation, as well as the critical role of NF-κB–NOD-, leucine-rich repeat and pyrin domain-containing protein 3 (NLRP3) inflammasome amplification in acute respiratory distress syndrome (ARDS) and sepsis-associated encephalopathy (SAE) [23, 24]. Concurrently, the rapid advancement of multi-omics technologies—such as single-cell transcriptomics, spatial omics, and time-series proteomic and metabolomic profiling—has facilitated the dynamic tracking of key pathway alterations throughout the course of sepsis [25]. By integrating these data, researchers can construct more accurate dynamic regulatory models using methodologies like DBNs, GNNs, and multi-scale network fusion (MSF), which facilitate the identification of network control hubs and bolster the development of system-level intervention strategies.

In contrast to many previous reviews that adopt a static perspective or concentrate on isolated signaling pathways, the present review prioritizes the dynamic evaluation of systemic toxicity in sepsis. By illustrating how signaling pathways evolve across different time points, immune states, and organs, this review proposes an innovative framework that connects network remodeling with multi-organ injury and cross-organ interactions. This narrative review focuses on the network reconstruction of systemic toxicity in sepsis, summarizing advancements in dynamic signaling pathways, organ-specific injury, inter-organ coupling, and multi-omics modeling. By integrating these dimensions, our work aims to provide readers with a novel systems-level perspective that enhances the understanding of disease heterogeneity and inspires precision-targeted strategies for sepsis management.

## Methods

This article presents a narrative review. We conducted a comprehensive search of PubMed, Web of Science, and Scopus for English-language publications from 2015 to 2025, utilizing combinations of the keywords “sepsis,” “systemic toxicity,” “multi-organ injury,” “network biology,” and “dynamic signaling pathways.” Additional references were identified by reviewing the bibliographies of relevant articles. We included studies that provided mechanistic insights into sepsis-associated systemic toxicity and multi-organ injury, while excluding case reports, conference abstracts, and non-mechanistic studies. A formal risk-of-bias assessment was not performed, as the objective of this review was to offer a narrative synthesis rather than a systematic appraisal of the evidence.

**Table 1:** Comparative table of sepsis-associated cell death mechanisms

**Type of cell death**	**Activation mechanism**	**Key pathways molecular signals**	**Functional implications**
Apoptosis [38] (programmed cell death)	Fas/FasL activation, cytochrome c release, caspase-3/9 activation	Bcl-2↓, caspase-3/9↑	Immune cell loss and immunoparalysis
Necrosis [39] (unregulated cell death)	Hypoxia, energy depletion, membrane rupture	HMGB1↑, extracellular ATP↑ → TLR/NLRP3	Release of DAMPs, inflammation amplification
Pyroptosis [28, 29, 41]	NLRP3 inflammasome → caspase-1 → GSDMD cleavage	NLRP3↑, caspase-1↑, IL-1β ↑	Inflammatory amplification, ARDS/liver injury
Ferroptosis [43]	Iron overload, lipid peroxidation	Fe^2+^↑, *GPX4*↓, MDA↑, 4-HNE↑	Lipid peroxidation–mediated injury in heart/kidney
Necroptosis [42]	RIPK1/3 → MLKL	RIPK1↑, RIPK3↑, MLKL↑	Amplifies necrosis and immune activation

Note: ↑ indicates upregulation/increase; ↓ indicates downregulation/decrease. Abbreviations: Bcl-2: B-cell lymphoma 2; HMGB1: High mobility group box 1; ATP: Adenosine triphosphate; TLR: Toll-like receptor; NLRP3: NOD-like receptor family pyrin domain-containing 3; GSDMD: Gasdermin D; IL-1β: Interleukin-1 beta; ARDS: Acute respiratory distress syndrome; Fe^2+^: Ferrous iron; GPX4: Glutathione peroxidase 4; MDA: Malondialdehyde; 4-HNE: 4-hydroxynonenal; RIPK1: Receptor-interacting serine/threonine-protein kinase 1; RIPK3: Receptor-interacting serine/threonine-protein kinase 3; MLKL: Mixed lineage kinase domain-like protein; DAMPs: Damage-associated molecular patterns.

### Systemic toxicity mechanisms associated with sepsis

#### The inflammation–immune dysregulation network

One of the hallmark features of sepsis is an imbalanced immune response to infection, characterized by excessive inflammatory activation and progressive immunosuppression. These processes may occur at different stages of the disease or simultaneously across various tissues, creating a complex network of inflammation and immune dysregulation [26, 27]. In the early stages of sepsis, pathogen-associated molecular patterns (PAMPs) and damage-associated molecular patterns (DAMPs) activate pattern recognition receptors, such as Toll-like receptors (TLRs) and NOD-like receptors (NLRs). This activation rapidly initiates signaling cascades, including NF-κB, MAPK, and JAK-STAT pathways, resulting in a significant release of inflammatory cytokines (e.g., IL-1β, TNF-α, and IL-6) and leading to a cytokine storm driven by pro-inflammatory networks. Concurrently, the activation of the NLRP3 inflammasome exacerbates both local and systemic inflammation by inducing pyroptosis and other forms of programmed cell death [28, 29]. At this stage, the signaling network demonstrates high centrality, redundancy, and significant pathway cross-talk, creating a tightly coupled inflammatory module marked by multi-pathway synergy and positive feedback amplification. Although pro-inflammatory mechanisms are essential for antimicrobial defense, their dysregulation can result in severe tissue damage and the exacerbation of systemic toxicity [30, 31].

During the progression of sepsis, the host activates anti-inflammatory responses to mitigate excessive immune activation [1]. However, this feedback mechanism is frequently overactivated, resulting in immunoparalysis. This condition is characterized by features such as T cell exhaustion, reduced expression of HLA-DR on monocytes, impaired antigen presentation, and the upregulation of immune checkpoint molecules, including PD-1 and cytotoxic T-lymphocyte-associated protein 4 (CTLA-4) [32, 33]. The sustained release of anti-inflammatory cytokines, including IL-10 and TGF-β, alongside elevated levels of regulatory T cells (Tregs), indicates a shift in the immune system from an activated to a dysfunctional state [34]. Research demonstrates that this immunosuppression does not simply occur as a consequence of inflammation; rather, it arises concurrently, establishing a state of “inflammation–immunosuppression coexistence” [9, 26]. In sepsis, inflammation and immune dysregulation involve dynamic shifts in signaling networks. Initially, pro-inflammatory pathways, such as NF-κB and MAPK, predominate; however, as the disease progresses, these pathways transition into immunosuppressive modules, including signal transducer and activator of transcription 3 (STAT3), IL-10, and PD-1, indicating a temporal evolution in the immune response. This shift can be quantified through network parameters, such as changes in centrality and reduced pathway efficiency. Spatial heterogeneity is observed across different tissues: lung inflammation is primarily driven by neutrophil infiltration and NLRP3 activation, whereas antigen presentation and T cell apoptosis are compromised earlier in the liver, spleen, and lymph nodes. Consequently, inflammation and immune dysregulation represent a temporally evolving and spatially heterogeneous network that contributes to systemic toxicity and organ dysfunction in sepsis [35].

#### Signaling pathways of apoptosis, necrosis, and regulated necrosis

Cell death plays a pivotal role in systemic toxicity during sepsis, serving not only as a result of tissue injury but also as a significant driver of inflammation, immune dysregulation, and multi-organ dysfunction [36, 37]. Initial research concentrated on classical apoptosis, characterized by Fas/FasL and TNF receptor signaling, as well as mitochondrial cytochrome c release, which activate caspase-3 and caspase-9. This cascade leads to extensive apoptosis of immune cells, including T cells, B cells, and dendritic cells, ultimately impairing host immune responses and contributing to immunoparalysis [38]. At the network level, this process is characterized by synchronized apoptosis among immune cell populations, downregulation of anti-apoptotic factors such as Bcl-2, and upregulation of pro-apoptotic receptors, collectively establishing a stable and efficient immune exhaustion module. Concurrently, conditions such as hypoxia, energy metabolism disorders, and membrane disruption associated with sepsis can induce non-programmed necrosis, leading to the release of intracellular contents like HMGB1 and ATP. This release subsequently activates inflammasomes and TLRs, triggering a toxic response mediated by overflow [39]. In recent years, the concept of regulated necrosis has significantly enhanced our understanding of the cell death network, incorporating novel pathways such as pyroptosis, necroptosis, and ferroptosis, which act as crucial links between inflammation and metabolic dysregulation (Table 1) [40].

Pyroptosis is defined by NLRP3 inflammasome-mediated caspase-1 activation and gasdermin D (GSDMD) cleavage, primarily occurring in neutrophils and macrophages, and is significantly implicated in ARDS and liver injury [41]. Necroptosis, driven by the RIPK1/RIPK3/MLKL signaling axis, typically occurs following caspase-8 inactivation and is closely associated with tissue necrosis across various organs [42]. Ferroptosis, induced by iron accumulation and lipid peroxidation, constitutes a critical mechanism of injury in metabolically active cells, such as cardiomyocytes and renal tubular epithelial cells [43]. These cell death pathways can be activated independently or synergistically, forming an interconnected network; for example, pyroptosis and necroptosis are often sequentially activated within the same cell, thereby amplifying inflammatory responses. Moreover, cell death pathways are regulated not only by inflammatory mediators but also by reciprocal activation of inflammatory signaling cascades, including NF-κB and STAT3, which establishes a feedback loop of “cell death–inflammation amplification–systemic toxicity propagation” [44].

From a dynamic network perspective, cell death signaling pathways in sepsis exhibit both temporal staging and organ-specific spatial characteristics [45]. For instance, apoptosis predominates in immune cells during the early phases, while pyroptosis and ferroptosis become more prevalent in parenchymal cells at later stages, indicating a progressive shift in dominant signaling nodes throughout disease evolution [46]. By integrating these mechanisms, a network model of cell death-related pathways can be developed to identify key cross-regulatory nodes (e.g., RIPK3, GSDMD, and *GPX4*) as potential targets for systemic toxicity intervention, thereby providing a theoretical framework for multi-organ protection strategies.

#### Oxidative stress and mitochondrial dysfunction

Oxidative stress is a crucial pathological component of systemic toxicity in sepsis, encompassing multiple phases, including inflammatory activation, immune regulation, cell death, and multi-organ dysfunction [47]. It functions as a central and dynamically active module within the sepsis signaling network. Characterized by an excessive accumulation of reactive oxygen species (ROS) and reactive nitrogen species (RNS) that exceed the capacity of antioxidant systems (e.g., superoxide dismutase [SOD], glutathione [GSH], and glutathione peroxidase [GPx]), oxidative stress leads to molecular damage and signaling dysregulation [48]. Mitochondrial dysfunction significantly contributes to this process by impairing ATP production, disrupting calcium homeostasis, and further enhancing ROS generation, thereby accelerating toxicity across multiple organs. Rather than viewing oxidative stress, inflammation, and cell death as distinct events, it is more accurate to consider them as an integrated pathogenic circuit. ROS activate NF-κB, MAPK, and NLRP3 inflammasome signaling while simultaneously inducing ferroptosis through lipid peroxidation and GPX4 inhibition, thus creating a self-reinforcing loop of “oxidative stress-inflammation-cell death [49].”

Organ-specific characteristics are evident: pulmonary injury is marked by NADPH oxidase-driven ROS bursts, whereas cardiac and renal tissues exhibit particular vulnerability to mitochondrial collapse. Modern omics technologies and network modeling offer significant opportunities to elucidate the mechanisms underlying mitochondrial function. The quantitative tracking of mitochondrial activity is now achievable through single-cell metabolomics and spatial transcriptomics. Additionally, graph-based algorithms, such as PageRank and network path analysis, facilitate the identification of regulatory bottlenecks in ROS signaling. Targeting antioxidant pathways, including Nrf2, GPX4, and SIRT3, has demonstrated potential in alleviating oxidative stress-induced organ damage; however, further validation of these approaches is necessary [50].

### Network-based dynamic evolution mechanisms of multi-organ injury

In addition to organ-specific injuries, recent studies emphasize that systemic toxicity in sepsis is mediated by molecular carriers that transmit signals across distant organs. DAMPs, such as HMGB1 and extracellular ATP, along with exosome-derived microRNAs and metabolic by-products (e.g., bile acids and lactate), play crucial roles as messengers in this process [39, 43]. For instance, exosomes released from inflamed pulmonary tissue can transfer miRNAs that upregulate TLR4 in renal tubular epithelial cells, thereby exacerbating acute kidney injury (AKI) [18]. Similarly, hepatic HMGB1 and bile acid metabolites are implicated in myocardial dysfunction and pulmonary inflammation, establishing a liver–heart axis of injury [36, 37]. These inter-organ messengers facilitate local injuries to trigger systemic amplification loops, converting organ-specific damage into multi-organ dysfunction [31].

#### Pulmonary injury: ARDS and disruption of the alveolar-capillary barrier

In sepsis, ARDS represents one of the earliest and most common forms of organ dysfunction, primarily resulting from significant disruption of the alveolar–capillary barrier [24]. This barrier, comprised of alveolar epithelial cells, capillary endothelial cells, and the basement membrane, is crucial for effective pulmonary gas exchange. In ARDS, its integrity is compromised due to complex immune and inflammatory responses, resulting in increased permeability, alveolar edema, and the formation of hyaline membranes [51].

Neutrophil recruitment and hyperactivation are critical in the early stages of lung injury, primarily through the release of elastase, myeloperoxidase (MPO), ROS, and the formation of neutrophil extracellular traps (NETs), which compromise alveolar cell integrity. Alveolar macrophages identify PAMPs and DAMPs via the TLR4–MyD88–NF-κB signaling pathway, leading to the secretion of pro-inflammatory cytokines such as IL-1β, IL-6, and TNF-α. This process amplifies the inflammatory response and facilitates the recruitment of additional immune cells.

Key signaling pathways, including NF-κB, MAPK, JAK/STAT, PI3K-AKT, and the NLRP3 inflammasome, orchestrate cytokine expression, pyroptosis, and disruption of the pulmonary barrier. The NF-κB–CXCL8–neutrophil axis and the NLRP3–GSDMD–IL-1β pathway are pivotal in mediating the cytokine storm associated with lung injury. Furthermore, the downregulation of tight junction proteins, such as ZO-1 and VE-cadherin, along with cytoskeletal remodeling through RhoA/ROCK and Src kinases, contributes to barrier breakdown.

Mitochondrial dysfunction and ROS accumulation further activate the NLRP3 inflammasome, establishing a feedback loop characterized by oxidative stress, pyroptosis, cytokine release, and barrier disruption. ARDS evolves from a predominantly pro-inflammatory response to a mixed pro- and anti-inflammatory network, ultimately progressing towards fibrosis. As a sentinel organ, the lung influences distant organs, including the kidneys, liver, and heart, underscoring the necessity for integrated multi-organ protection strategies. Distinct from renal injury, pulmonary damage is primarily driven by barrier disruption mediated by the NF-κB–NLRP3–NETs axis, emphasizing the lung’s unique role as the “first responder” in systemic toxicity.

#### Renal injury: AKI

In sepsis, AKI is the most prevalent organ dysfunction following ARDS, with an incidence exceeding 50%. It is strongly correlated with increased mortality and the progression to chronic kidney disease. Sepsis-associated AKI (SA-AKI) is now understood not only as a consequence of hypoperfusion but also as a manifestation of systemic toxicity driven by inflammation, metabolic dysregulation, and programmed cell death [52]. The pathogenesis of SA-AKI begins with the activation of pro-inflammatory pathways, such as NF-κB and JAK/STAT, via pattern recognition receptors, including TLR4 and NOD1/2. This activation leads to the release of inflammatory mediators such as IL-6, IL-1β, and MCP-1 from tubular epithelial cells, promoting the infiltration of neutrophils and macrophages and amplifying local inflammation. Renal immune dysregulation, characterized by a predominance of M1 macrophages and sustained activation of inflammatory cytokines, contributes to a tightly coupled feedback network [20]. The mechanisms of cell death in SA-AKI are complex, involving apoptosis (caspase-3/9 activation), pyroptosis (NLRP3-caspase-1-GSDMD axis), and ferroptosis (*GPX4* inhibition and lipid peroxidation). These pathways lead to renal tubular damage and the release of DAMPs, which further amplify inflammation, creating a toxic feedback loop of cell death, inflammation, and microenvironmental collapse.

Oxidative stress and mitochondrial dysfunction also play critical roles in SA-AKI, characterized by increased mitochondrial ROS production, suppressed Nrf2 antioxidant signaling, and dysregulated Keap1 activation that drive ferroptosis and metabolic disturbances. Additionally, a shift from oxidative phosphorylation to glycolysis in tubular cells disrupts ATP production and Na^+^/Ca^2+^ transport.

**Figure 1.**
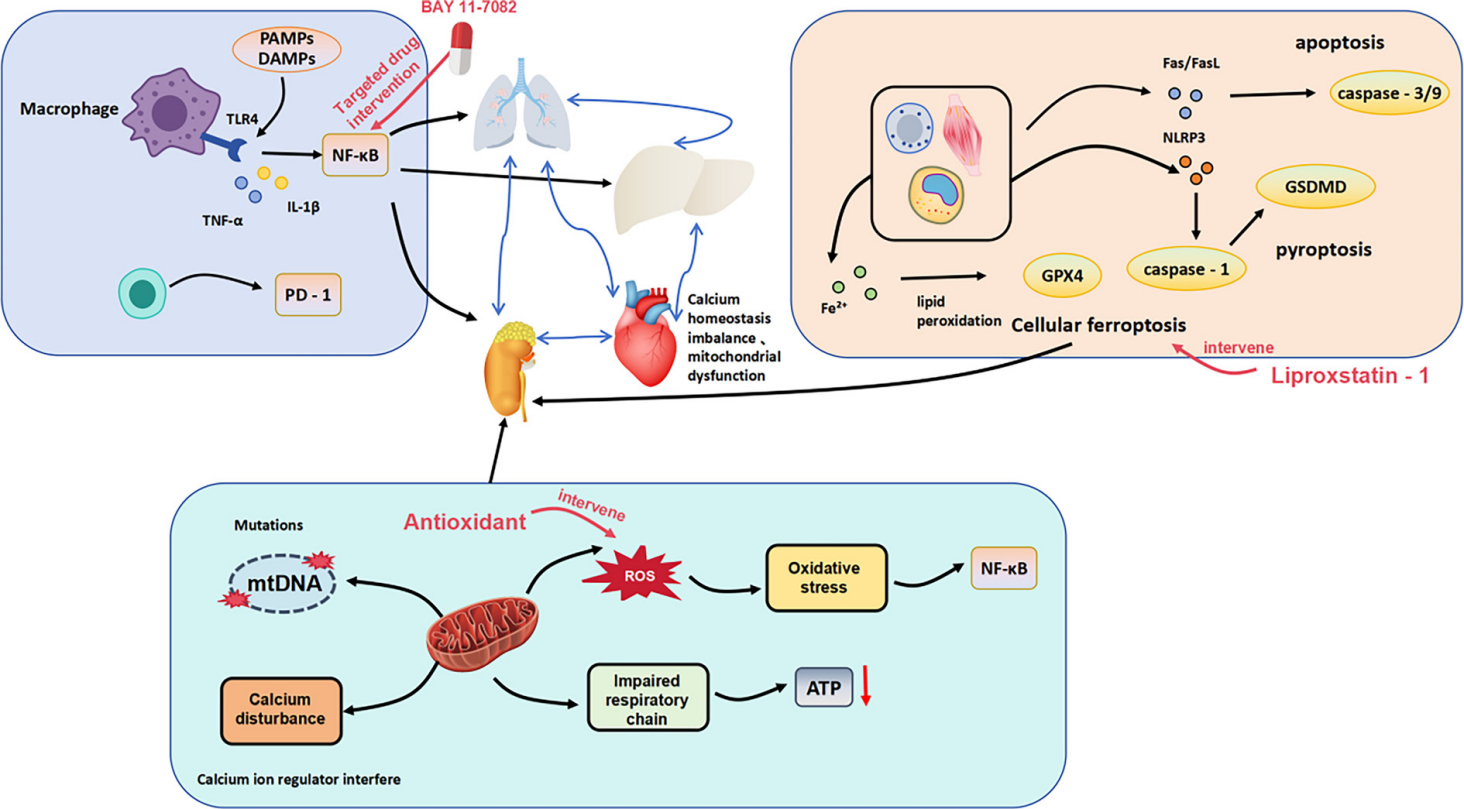
**Schematic representation of the systemic toxicity network in sepsis-associated acute kidney injury (SA-AKI).** The figure shows the interconnected feedback loops among kidney, lung, liver, and heart. Pathways illustrated include inflammatory signaling (NF-κB, cytokines), mitochondrial dysfunction (mtDNA mutations, ROS, ATP depletion), and programmed cell death mechanisms (apoptosis, pyroptosis, ferroptosis). Therapeutic interventions (BAY 11-7082, Liproxstatin-1) are indicated. Arrow colors: red = inflammatory amplification, blue = metabolic dysregulation, green = therapeutic modulation. Abbreviations: PAMPs: Pathogen-associated molecular patterns; DAMPs: Damage-associated molecular patterns; TLR4: Toll-like receptor 4; TNF-α: Tumor necrosis factor alpha; IL: Interleukin; PD-1: Programmed cell death protein 1; NF-κB: Nuclear factor kappa-light-chain-enhancer of activated B cells; mtDNA: Mitochondrial DNA; ROS: Reactive oxygen species; ATP: Adenosine triphosphate; GPX4: Glutathione peroxidase 4; Fas/FasL: Fas receptor/Fas ligand; NLRP3: NOD-, LRR- and pyrin domain-containing protein 3; GSDMD: Gasdermin D.

SA-AKI progresses from inflammation-driven signaling to metabolic imbalance and cell death, with shifts in central network nodes and reconfigured modules. Cross-organ inflammation, particularly between the lung–kidney and liver–kidney axes, exacerbates renal injury, as evidenced by IL-6 and exosomal miRNAs upregulating TLR4 in renal tubules. Overall, SA-AKI represents a systemic network pathology, as illustrated in Figure 1. This renal pathology contrasts with pulmonary injury by emphasizing multi-modal cell death and metabolic imbalance as central drivers.

This figure depicts the interconnected toxicity feedback loop among the kidney, lung, liver, and heart. Key pathways include the NF-κB/JAK-STAT–IL-6/IL-1β signaling cascade [52], the NLRP3–caspase-1–GSDMD pyroptosis axis [29], and ferroptosis mediated by *GPX4* inhibition [43]. (Arrow colors: red = inflammatory amplification, blue = metabolic dysregulation, green = therapeutic modulation.) Drug annotations include: BAY 11-7082 (NF-κB inhibitor, 5–20 µM) [11], MCC950 (NLRP3 inhibitor, 10 µM) [29], disulfiram (GSDMD inhibitor, 1–10 µM) [29], and *GPX4* agonists (ferroptosis protection) [43].

These pathways and pharmacological agents represent potential targets for intervention within the systemic toxicity network.

#### Hepatic injury and metabolic dysregulation

Throughout the progression of sepsis, the liver, recognized as a central immunometabolic organ, is frequently impacted by systemic toxicity at an early stage [36, 37, 53]. Hepatic dysfunction is characterized not only by elevated transaminases and bilirubin abnormalities but also by a more intricate network-level interaction among inflammatory, metabolic, and cell death pathways.

During the inflammatory activation phase, Kupffer cells identify PAMPs and DAMPs through receptors such as TLR4 and RAGE. This recognition activates NF-κB and JAK/STAT signaling pathways, resulting in the release of pro-inflammatory cytokines TNF-α and IL-6. Consequently, the NLRP3 inflammasome-mediated pyroptosis occurs, leading to damage in hepatocytes and sinusoidal endothelial cells, which contributes to microcirculatory dysfunction and regional hypoxia. Concurrently, hepatocellular energy metabolism undergoes reprogramming characterized by mitochondrial dysfunction, disruption of the tricarboxylic acid (TCA) cycle, and impaired ATP production, all of which contribute to metabolic stress. The suppression of nuclear receptors such as PPARα and FXR disrupts bile acid homeostasis, further exacerbating cellular injury [54]. Additionally, the accumulation of lipid peroxidation products, such as malondialdehyde (MDA) and 4-hydroxynonenal (4-HNE), activates ferroptosis pathways, creating a synergistic toxic circuit that links inflammation, metabolic disturbances, and cell death.

Septic liver injury is characterized by dynamic modular reconfiguration and alterations in signaling pathways. Initially, the NF-κB-driven pro-inflammatory module predominates, followed by the activation of NLRP3-pyroptosis and PPARα-metabolic modules during the intermediate stage. Subsequently, the process transitions to immunoregulatory pathways involving IL-10 and TGF-β. Key regulatory nodes, such as SIRT1, Nrf2, and *GPX4* orchestrate these transitions. Moreover, the liver serves as a source of inflammatory mediators, exosomes, and metabolic products that influence distant organs, including the lungs, heart, and kidneys, thereby establishing a cross-organ signaling network. Thus, septic liver injury represents a systemic network process shaped by inflammation, metabolic disruption, and cell death, highlighting its potential for multi-organ protection and precision therapy.

#### Myocardial injury and microcirculatory dysfunction

Septic cardiomyopathy (SCM) is a functional cardiac disorder influenced by various factors, including inflammation, microcirculatory impairment, mitochondrial dysfunction, and disruptions in calcium homeostasis [55]. During sepsis, inflammatory cytokines such as TNF-α, IL-1β, and IL-6 activate signaling pathways like NF-κB, JAK/STAT, and MAPK through cardiomyocyte membrane receptors. This activation leads to the release of myocardial depressant factors, dysregulation of calcium channels, and programmed cell death. Additionally, local immune cell infiltration and complement activation exacerbate myocardial inflammation, promoting apoptosis and contractile dysfunction.

At the microvascular level, endothelial injury and an imbalance of vasoactive substances (e.g., nitric oxide and endothelin) result in heterogeneous myocardial perfusion, further contributing to metabolic derangement and oxidative stress. Mitochondrial dysfunction in cardiomyocytes is characterized by a loss of membrane potential, opening of the mitochondrial permeability transition pore (mPTP), and excessive release of ROS. These factors collectively inhibit ATP production and activate the caspase cascade, leading to cell death [56]. Concurrently, calcium dysregulation worsens the injury, with Ca^2+^ overload impairing myofilament contraction and triggering calpain-mediated cytoskeletal degradation.

The signaling evolution of SCM reflects dynamic remodeling, shifting from inflammation-dominated pathways to those focused on metabolism and cell death. Early dominant signals such as NF-κB and STAT3 are progressively supplanted by pathways involving ROS, mPTP, and Nrf2, culminating in the late-stage activation of reparative modules like TGF-β and vascular endothelial growth factor (VEGF). Multiple pathways converge on shared regulatory nodes (e.g., NF-κB, iNOS, and *GPX4*), creating a high-density toxic module characterized by dynamically shifting signal intensity and centrality throughout disease progression.

Furthermore, cardiac dysfunction exacerbates damage to other organs through hypoperfusion and circulatory instability, leading to complications such as AKI and intestinal barrier breakdown. Simultaneously, pulmonary inflammatory mediators can disseminate through the bloodstream to the myocardium, triggering localized inflammatory responses and establishing a typical inter-organ toxic loop. Therefore, SCM represents a complex systemic toxicity network shaped by inflammation, microcirculatory disruption, and metabolic collapse. A comprehensive understanding of its evolutionary trajectory and network remodeling mechanisms may reveal novel therapeutic targets for multi-organ protection.

**Figure 2.**
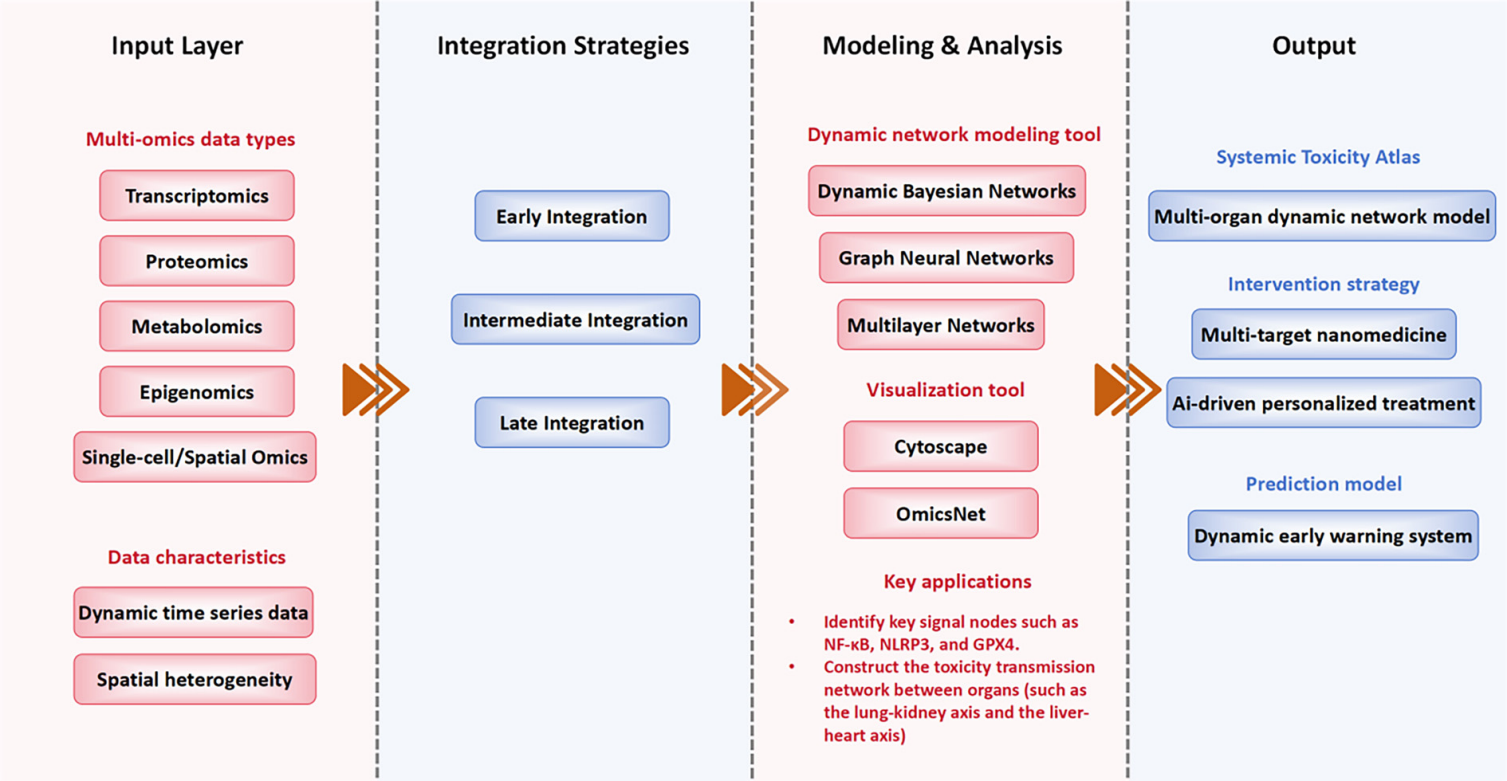
**Computational frameworks for multi-omics integration and dynamic network reconstruction in systemic toxicity of sepsis.** The figure shows input omics layers, integration strategies, modeling and visualization tools, as well as key applications and resulting outputs. Abbreviations: DBN: Dynamic Bayesian network; GNN: Graph neural network; NF-κB: Nuclear factor kappa-light-chain-enhancer of activated B cells; NLRP3: NOD-, LRR- and pyrin domain-containing protein 3; GPX4: Glutathione peroxidase 4.

### Methods and tools for reconstructing systemic toxicity networks

#### Integrated analysis strategies for multi-omics data

The systemic toxicity of sepsis encompasses the coordinated evolution of multiple biological systems and pathways, including inflammation, immunity, metabolism, and regulated cell death. This phenomenon is characterized by considerable complexity, temporal dynamics, and inter-individual heterogeneity. Traditional single-omics approaches prove inadequate in capturing this complexity; consequently, multi-omics integration has emerged as a pivotal strategy for elucidating the underlying mechanisms of network remodeling [57, 58]. Common omics layers, including transcriptomics, proteomics, metabolomics, epigenomics, and single-cell/spatial omics, capture distinct biological dimensions such as gene expression, protein translation, metabolism, and cellular heterogeneity. Integration strategies can be categorized into three types: early integration, which merges data for joint dimensionality reduction; intermediate integration, which models each omic layer independently before aligning them biologically through pathways or co-expression networks; and late integration, which analyzes heterogeneous datasets after individual modeling. Analytical tools like iCluster, MOFA, and SNF facilitate the reduction of multi-omics features, while Cytoscape and OmicsNet support network visualization. Additionally, GNNs and Bayesian modeling are effective for inferring dynamic biological processes [59]. In the context of sepsis, multi-omics studies elucidate the divergent signaling pathways of NF-κB and NLRP3 across various organs, underscoring the heterogeneity of inflammatory networks. Integrative analyses of metabolomics and proteomics demonstrate the role of *GPX4*-mediated ferroptosis in both renal and myocardial tissues. Although multi-omics integration facilitates the construction of regulatory networks and the identification of critical signaling pathways, it encounters challenges related to data heterogeneity and computational complexity. Innovative methodologies, including causal inference and spatial transcriptomics, hold promise for enhancing biological resolution [60]. Multi-omics and dynamic evaluation have been extensively applied in various diseases. In oncology, for instance, longitudinal single-cell and spatial transcriptomics have been employed to elucidate tumor evolution and therapeutic resistance [61]. In neurodegenerative disorders, such as Alzheimer’s disease, time-series metabolomics and proteomics have uncovered progressive mitochondrial dysfunction and synaptic loss [62]. In cardiovascular research, integrative omics combined with network modeling has identified the dynamic lipid metabolism and immune-inflammatory interactions that drive the progression of atherosclerosis [63]. In contrast to these studies, our review provides a distinct perspective on sepsis by focusing on multi-organ, cross-system coupling and the network reconstruction of systemic toxicity, rather than relying on static or organ-restricted models. Section 5.2 details computational modeling frameworks for multi-omics integration. Overall, multi-omics offers critical insights into the dynamic signaling evolution and network remodeling associated with sepsis toxicity (Figure 2).

This figure illustrates the computational methods employed to integrate heterogeneous omics data for network reconstruction. The methodologies include weighted gene co-expression network analysis (WGCNA) [64], DBNs [25], and GNNs [57]. The arrow colors denote the following: black indicates the data integration flow, while orange signifies iterative refinement using artificial intelligence (AI) models.

Node colors are categorized as follows: yellow represents key signaling pathways (e.g., NF-κB, NLRP3, and *GPX4*), blue denotes metabolic regulators, and purple indicates immune checkpoints.

AI-driven models demonstrate the application of machine learning and deep learning techniques, such as GNNs and recurrent neural networks, to capture dynamic and cross-organ regulatory relationships.

#### Network modeling and dynamic simulation methods

Building upon the multi-omics integration strategies discussed in Section 5.1, network modeling serves as an analytical framework to reconstruct the dynamic interactions underlying sepsis. Early static models, including protein–protein interaction (PPI) networks, co-expression networks (such as WGCNA), and transcription factor–miRNA–target gene networks, have enhanced our understanding of structural interactions in sepsis [64]. However, these models do not adequately capture the dynamic changes that occur during disease progression.

To address these limitations, advanced modeling approaches—such as DBNs, ordinary differential equation (ODE) frameworks, Boolean networks, and GNNs—have been developed to infer temporal signaling dynamics and cross-organ interactions. Strategies for modeling multi-organ injury, including multilayer networks, cell–cell communication tools (for instance, CellChat), and tissue-specific networks, have facilitated the investigation of signal coupling and toxicity across organs. Notable findings from these models include the identification of the STAT3–iNOS module within myocardial toxicity networks.

Despite challenges such as data heterogeneity and temporal resolution, the integration of omics, spatiotemporal data, and AI-driven models holds promise for enhancing predictive and intervention strategies in sepsis (see Table 2).

**Table 2:** Comparative analysis of modeling approaches

**Model types**	**Representative methods**	**Modeling features**	**Applicable scenarios**	**Advantages**	**Challenges**
Static networks [57, 64]	PPI, WGCNA, TF–miRNA	Interaction-based network construction; regulatory relationship inference	Pathway co-expression; transcriptional regulation inference	Clear structure, suitable for early screening	Inability to simulate time variation; weak dynamic prediction
Dynamic Bayesian networks [45]	DBN	Node states vary over time; sequence-based modeling	Pathway activation order, signal propagation dynamics	Capable of handling incomplete data; supports temporal inference	High computational complexity; time-dependent data labeling required
ODE-based systems [45]	ODE frameworks	Continuous modeling of dynamic transitions	Biochemical kinetics, pathway activity prediction	High quantitative resolution; mechanistic interpretability	Requires large prior parameter sets; sensitive to data quality
Boolean networks [45]	Boolean network	Binary-state modeling of on/off mechanisms	Logical state transition analysis	Simple structure, suitable for low-data or multi-state systems	Difficult to model continuous transitions; lacks quantitative expressiveness
Graph neural networks [25, 57]	GCN, GAT, Hetero-GNN	High-dimensional graph learning; inter-organ/multi-omic integration	Multi-organ signaling network integration	Strong nonlinear modeling capacity; adaptable to complex systems	Requires large datasets; interpretability may be limited
Multilayer/cross-organ networks [25, 57]	CellChat, tissue-GNN	Integrates cell–cell, tissue–organ, and spatial layers	Signal cross-talk, spatially resolved organ interaction networks	Captures cross-scale and spatial interactions; supports spatial modeling	High data demands; model complexity and parameter tuning required

Abbreviations: PPI: Protein–protein interaction; WGCNA: Weighted gene co-expression network analysis; TF: Transcription factor; miRNA: MicroRNA; DBN: Dynamic Bayesian network; ODE: Ordinary differential equation; GCN: Graph convolutional network; GAT: Graph attention network; GNN: Graph neural network.

A direct comparison of these modeling strategies underscores their unique applicability in capturing the temporal-spatial complexity of sepsis. DBNs are especially beneficial for inferring causal activation orders of signaling pathways in the presence of incomplete data. However, their high computational demands and the need for finely resolved time-series data restrict their use in large-scale applications [65].

ODE-based frameworks provide robust mechanistic interpretability and high-resolution simulations of biochemical kinetics; however, they necessitate extensive prior knowledge of parameters and are highly sensitive to data quality. In contrast, Boolean networks are computationally efficient and suitable for exploratory analyses in low-data contexts, but they only facilitate binary state transitions, limiting their ability to model graded or continuous molecular dynamics [66]. GNNs excel at integrating heterogeneous multi-omics and multi-organ data, allowing for the reconstruction of high-dimensional toxicity networks characterized by nonlinear interactions. Nonetheless, GNNs require large datasets and complex training processes, which may compromise their interpretability compared to mechanistic models [67]. Collectively, these considerations indicate that no single method is universally optimal; therefore, hybrid or multi-model strategies may be the most effective approach for modeling sepsis toxicity networks.

### Therapeutic target identification and prospects for precision intervention

#### Research progress in systemic toxicity intervention strategies

Sepsis-induced systemic toxicity arises from the dysregulation of multiple signaling pathways, cell death, metabolic imbalance, and immune dysfunction. Consequently, therapeutic strategies are transitioning from traditional anti-inflammatory and organ support approaches to systemic regulation that targets key network nodes. At the signaling level, NF-κB, JAK/STAT, and the NLRP3 inflammasome are critical targets [68]. Small-molecule inhibitors such as BAY 11-7082, ruxolitinib, and VX-765 block pro-inflammatory pathways, thereby reducing the risk of multi-organ injury. Recent advances in targeting programmed cell death, particularly pyroptosis and ferroptosis, have led to the development of agents like MCC950, disulfiram, and *GPX4* agonists that disrupt the inflammation–cell death cycle [69]. Additionally, metabolic reprogramming through Nrf2 activation or AMP-activated protein kinase (AMPK) agonists enhances antioxidant capacity, mitochondrial function, and energy metabolism, mitigating organ dysfunction [70]. Immune reconstitution strategies, including IL-7 supplementation and PD-1/PD-L1 blockade, aim to restore T cell function and improve antigen presentation [71]. In summary, these therapeutic advancements underscore the significance of pathway-specific and multi-target interventions for systemic detoxification and organ protection.

#### Personalized medicine and dynamic early warning systems

Beyond pathway-targeted therapies, personalized medicine plays a crucial role in addressing the heterogeneity of sepsis patients. By integrating multi-omics data—including transcriptomics, proteomics, metabolomics, single-cell RNA sequencing, and spatial transcriptomics—a comprehensive “systemic toxicity atlas” can be developed for each individual patient [72]. This atlas facilitates the mapping of critical signaling pathways, including NF-κB, NLRP3, JAK/STAT, and *GPX4* across various organs, thereby identifying individualized toxicity network hubs. In contrast to conventional static systems such as SOFA, time-series models—specifically DBNs, GNNs, and RNNs—provide enhanced accuracy in predicting complications such as ARDS, AKI, and MODS [73]. AI platforms can integrate electronic medical records, real-time monitoring data, and omics profiles to dynamically guide interventions [74]. The development of digital twins facilitates the creation of in silico patient-specific models that simulate therapeutic outcomes and enable closed-loop treatment adjustments [73, 74]. Collectively, these strategies advance sepsis care toward precision-driven, mechanism-based management, complementing the therapeutic advances discussed in Section 6.1.

### Limitations

This narrative review presents several limitations that must be acknowledged. First, as a non-systematic review, there is an inherent risk of selection bias in the studies cited, despite our efforts to encompass the most pertinent literature. Second, the synthesis predominantly relies on secondary data from published reports, which may be subject to methodological heterogeneity and varying quality. Third, the primary studies included exhibit significant heterogeneity in experimental models, patient populations, and analytical approaches, limiting the direct comparability of findings. Finally, the discussion surrounding AI-driven “toxicity atlases” and predictive modeling remains speculative at this stage, necessitating further empirical validation prior to clinical application. These limitations underscore the necessity for cautious interpretation of our conclusions and indicate that the concepts presented should be viewed as hypothesis-generating rather than definitive.

In addition to these methodological considerations, critical translational challenges warrant attention. Organ-specific drug delivery obstacles continue to hinder the efficacy of pathway modulators, while potential off-target effects and adaptive resistance mechanisms may compromise long-term outcomes. At the clinical level, variability among patient populations, regulatory requirements, and the absence of standardized implementation protocols present additional barriers that complicate bedside application. Furthermore, although AI-driven warning systems are conceptually promising, they require high-quality, large-scale, and interoperable datasets; issues related to interpretability, real-time data integration, and rigorous clinical validation remain unresolved. Collectively, these translational barriers highlight that while systemic toxicity network-based interventions possess significant potential, substantial work remains necessary before they can be safely and effectively integrated into sepsis care.

## Conclusion

Sepsis, as a systemic disease, is not driven solely by inflammation or perfusion deficits but rather by a system-wide toxicity network reconstruction process involving inflammation, immune dysregulation, metabolic disturbances, programmed cell death, and multi-organ dysfunction. With advancements in multi-omics technologies, network biology, and dynamic modeling, researchers have progressively elucidated the spatiotemporal evolution of key signaling pathways—including NF-κB, JAK/STAT, NLRP3, and *GPX4*—across different organs, resulting in the construction of a comprehensive toxicity map characterized by multi-pathway, multi-node, and multi-organ coupling. This review takes systemic toxicity as a central framework to comprehensively summarize the mechanisms of signaling pathway remodeling, organ injury evolution, and network modeling strategies. It further outlines recent advances in therapeutic interventions including pathway modulation, regulation of cell death, metabolic reprogramming, and immune remodeling. In addition, we highlight the potential of multi-omics-driven personalized toxicity mapping, AI-assisted risk prediction models, and closed-loop feedback control systems in achieving individualized precision medicine. Future research should focus on multidimensional data integration, causal graph modeling, cross-organ network prediction, and digital twin technologies to advance systemic toxicity from mechanistic understanding to controllable modulation. In conclusion, this narrative review provides a systemic toxicity–centered perspective that offers a more comprehensive understanding of the pathophysiology of sepsis, laying a solid foundation for multi-organ protection and precision therapy. However, further research is needed to address the challenges of integrating multi-omics data and refining intervention strategies. Marking a critical transition in critical care medicine toward systematization, personalization, and intelligent management.

## Data Availability

The data used to support the findings of this study are available from the corresponding author upon request.
